# AI-Enhanced Virtual LIG–IoT Sensor Framework for Microclimatic Stress Prediction in *Vasconcellea stipulata* (Toronche) from Southern Ecuador

**DOI:** 10.3390/s26061766

**Published:** 2026-03-11

**Authors:** Alan Cuenca-Sánchez, Fernando Pantoja-Suárez

**Affiliations:** 1 Escuela de Formación de Tecnólogos, Escuela Politécnica Nacional, Quito 170143, Ecuador; 2 Departamento de Materiales, Escuela Politécnica Nacional, Quito 170143, Ecuador; fernando.pantoja@epn.edu.ec

**Keywords:** artificial intelligence, biodiversity conservation, environmental monitoring, graphene-based sensors, internet of things, laser-induced graphene, predictive modeling, *Vasconcellea stipulata* (toronche)

## Abstract

Microclimatic stress strongly influences the ecological resilience of *Vasconcellea stipulata* (Toronche), yet current monitoring approaches rely on sparse measurements and lack real-time predictive capability. This work introduces an AI-enhanced virtual sensing framework based on laser-induced graphene (LIG) designed to emulate the thermoresistive response of an LIG transducer and generate high-resolution environmental indicators for microclimatic analysis. Unlike conventional LIG sensors or standalone IoT systems, the proposed framework integrates experimental calibration, data-driven modeling, and embedded inference into a unified architecture suitable for lightweight deployment on edge devices. A multilayer perceptron (MLP) model trained on laboratory data reproduced the temperature- and humidity-dependent electrical behavior of the transducer with high fidelity, achieving an RMSE of 0.016 kΩ in the calibrated range (10–60 °C) and remaining below 0.09 kΩ under noisy and extrapolated conditions. Sensitivity analysis identified temperature as the dominant driver (71%), followed by solar irradiance (19%) and relative humidity (10%), consistent with the microstructural mechanisms governing LIG’s response. The virtual sensor enables continuous, low-cost environmental monitoring and provides quantitative variables that can support downstream ecological interpretation. Overall, the results highlight the potential of AI-enhanced LIG–IoT architectures for advancing real-time microclimatic assessment in resource-limited Andean ecosystems.

## 1. Introduction

The Andean high-altitude ecosystems of southern Ecuador represent one of the planet’s most fragile yet biologically diverse biomes, where anthropogenic pressures and climate anomalies are rapidly transforming ecological equilibria. Over recent decades, these montane systems have been increasingly affected by temperature anomalies, erratic precipitation, soil degradation, and the expansion of agricultural frontiers, leading to a progressive decline in endemic flora and fauna [1,2]. Within this ecological context, *Vasconcellea stipulata* (commonly known as “toronche”)—a native fruit tree of the family *Caricaceae*—embodies both the uniqueness and the vulnerability of Ecuador’s inter-Andean valleys. The species naturally grows between approximately 2000 m and 2600 m a.s.l., occupying transitional zones between dry montane scrublands and humid Andean forests across southern Ecuador and northern Peru [3,4]. Its morphological and physiological traits, including leafy stipules and tolerance to sub-freezing temperatures, reflect an evolutionary adaptation to high-altitude stressors [5]. However, its genetic structure is threatened by habitat fragmentation, uncontrolled hybridization, and the absence of ex situ germplasm conservation programs [6].

Despite its nutritional and industrial potential—its pulp contains carotenoids, phenolics, and enzymes similar to papain—*Vasconcellea stipulata* remains marginalized in both research and production systems [7]. The globalization of high-yield commercial crops has displaced traditional Andean cultivars, pushing the toronche toward ecological and cultural erosion. In Loja and surrounding regions, field observations suggest that populations are aging and spatially discontinuous, while regeneration rates in natural stands remain extremely low [8]. Beyond its nutritional value, the species holds cultural and ancestral significance for Andean communities. Traditionally cultivated in family orchards and used in artisanal foods and natural remedies, its gradual disappearance reflects both ecological and sociocultural vulnerability. This reinforces the urgency of developing conservation strategies that integrate technological innovation with the protection of local biodiversity and traditional knowledge (see Appendix A for a visual reference of the fruit).

Conservation practices based solely on field inventories or sporadic botanical registries are insufficient to address these emerging risks. Virtual sensing architectures that couple transducer models with AI-driven environmental datasets can help overcome this limitation by offering continuous and scalable observability in data-scarce regions. This underscores a pressing need for integrative monitoring frameworks that combine ecological knowledge, remote sensing, and artificial intelligence to predict environmental stressors before local extinction thresholds are reached [9,10].

In parallel, environmental sensing has entered a transformative phase driven by the convergence of microelectronics, nanomaterials, and intelligent data processing [11]. Recent advances in low-cost sensor fabrication and Internet of Things (IoT) architectures have enabled real-time environmental data acquisition in regions previously considered technologically isolated. Within this context, laser-induced graphene (LIG) has emerged as a promising material for next-generation sensors due to its high sensitivity, flexibility, and environmental sustainability. Produced through direct laser scribing of polymeric or lignocellulosic substrates, LIG forms a three-dimensional porous graphene network with excellent electrical conductivity, chemical stability, and mechanical resilience [12,13]. Unlike conventional graphene fabrication routes requiring high temperatures and toxic reagents, LIG production is single-step, solvent-free, and compatible with biodegradable materials, avoiding the use of hazardous chemicals and aligning with global sustainability goals [12,14].

LIG-based transducers have demonstrated strong performance in detecting gases such as NO_2_, NH_3_ and CO_2_; monitoring humidity and temperature; and sensing strain, pressure, or biochemical analytes [15,16,17]. Their tunable surface chemistry enables selective functionalization with metal nanoparticles or polymeric films, enhancing specificity for environmental analytes and pollutants [14,17]. Furthermore, the adaptability of LIG devices for flexible or wearable configurations has catalyzed their integration into IoT-based environmental and agricultural monitoring platforms [11,18]. Despite these advances, few studies have explored the potential of LIG sensors for ecological monitoring or biodiversity conservation in mountainous and remote ecosystems, where conventional instrumentation is often impractical due to cost, power constraints, or maintenance requirements [13,16]. Importantly, no prior work has integrated an LIG-based thermoresistive model into a fully virtual AI-driven microclimatic sensing framework for predictive ecological risk assessment.

Simultaneously, artificial intelligence (AI) and machine learning (ML) have transformed environmental analytics by enabling predictive pattern extraction from large, heterogeneous datasets. When combined with IoT architectures and open-access environmental databases—such as NASA POWER or INAMHI—AI models can reconstruct microclimatic trends and forecast habitat conditions under different climatic scenarios [19,20,21,22]. These databases, which integrate satellite-derived meteorological variables and downscaled projections, have been successfully validated for the Andean region [2,23]. In this context, the integration of simulated LIG-based transducers with AI-enhanced virtual sensing architectures represents an emerging frontier in data-driven biodiversity conservation [9,10,18]. Such systems can serve as “virtual ecological observatories,” capable of estimating temperature, humidity, and solar radiation dynamics across fragmented landscapes and supporting conceptual assessments of vulnerability in endemic species such as *Vasconcellea stipulata*.

Despite these advances, current research remains fragmented. Physical LIG sensors require laboratory fabrication and real-time instrumentation, while AI- and IoT-based ecological monitoring frameworks typically exclude material-level transducer modeling and the physical mechanisms governing sensor response. To date, no study has integrated an LIG-derived thermoresistive model into a fully virtual AI-enhanced microclimatic monitoring framework capable of operating in data-scarce Andean ecosystems. This gap limits the development of predictive, low-cost tools for ecological stress assessment in threatened species such as *Vasconcellea stipulata*. Accordingly, the central research question guiding this work is

*Can a virtual LIG-based sensing model, enhanced through AI, reliably emulate environmental responses relevant to microclimatic stress in resource-limited ecosystems?*

In contrast to existing studies—where LIG sensors are physically fabricated, calibrated through laboratory instrumentation, or deployed as isolated IoT nodes—the present work introduces a unified virtual framework that emulates the thermoresistive behavior of LIG, integrates open-access climatic variables, and processes them through an AI model embedded within an ESP32-oriented architecture. This combination is novel because it provides a fabrication-free, computationally lightweight approach for exploring how graphene-based sensing could operate within environmental monitoring systems without requiring physical prototypes or field deployments. By merging nanomaterial-level behavior, machine learning inference, and virtual IoT device simulation, the proposed framework contributes a methodological advance to the environmental sensing literature and expands the conceptual tools available for biodiversity monitoring in data-scarce mountainous ecosystems.

To further clarify the coherence between the study’s objectives and its outcomes, it is important to emphasize that the goal of this work is not to develop a high-capacity forecasting architecture, but rather to construct a physically grounded *virtual transducer* capable of reproducing the dominant thermoresistive mechanisms of LIG under realistic microclimatic variability. The practical significance of the model lies in its ability to generate continuous, high-resolution environmental indicators in regions where conventional instrumentation is limited, while maintaining computational efficiency and interpretability compatible with low-power IoT deployments. The results presented in this manuscript directly address this objective by demonstrating that a lightweight, physically interpretable neural architecture can successfully emulate LIG behavior, generalize across environmental conditions, and provide a methodological foundation for future ecological or hardware-oriented extensions.

The remainder of this manuscript is organized as follows. Section 2 describes the theoretical framework, datasets, and computational architecture used to construct the virtual sensing model. Section 3 presents the numerical results, including model convergence, robustness analyses, and sensitivity evaluation. Section 4 discusses the implications of the proposed framework for microclimatic monitoring and biodiversity conservation. Finally, Section 5 summarizes the main conclusions and outlines potential directions for future work.

## 2. Materials and Methods

### 2.1. Fabrication and Physical Characterization of the Laser-Induced Graphene (LIG) Sensor

The baseline sensor used in this study was fabricated following the laser-induced graphene (LIG) methodology validated by Cuenca-Sánchez et al. [24]. A 75 µm polyimide (PI) substrate was irradiated with a CO_2_ laser (10.6 µm wavelength, 60 W output power, 25 mm/s scan speed), inducing localized photothermal pyrolysis and forming a porous, conductive graphene network. The resulting structure exhibited strong adhesion to the substrate, mechanical flexibility and environmental stability.

Electrical contacts were applied using a silver-based conductive epoxy, and the device was encapsulated in acrylic to enhance mechanical protection and minimize oxidation. Raman spectroscopy confirmed the formation of graphenic carbon through the characteristic D (1340 ${\mathrm{cm}}^{-1}$), G (1580 ${\mathrm{cm}}^{-1}$), and 2D (2680 ${\mathrm{cm}}^{-1}$) bands. The measured $I_{2D}/I_{G}$ ratio of approximately 0.9 indicated a multilayer morphology favorable for thermal interaction and charge transport. Scanning electron microscopy (SEM) revealed an interconnected porous lattice that increases the effective surface area and supports enhanced thermoresistive sensitivity.

The fabricated device was experimentally characterized as a thermoresistive transducer. The electrical response exhibited a nominal resistance of 14.6 kΩ at 25 °C, with a temperature coefficient of −6.69 Ω/°C and a piezoresistive coefficient of −6.87 Ω/bar. Although the sensor demonstrates dual sensitivity to temperature and pressure, only its thermal response was considered in the present study, as variations in atmospheric pressure across the Andean highlands are negligible for the intended application.

**Figure 1:**
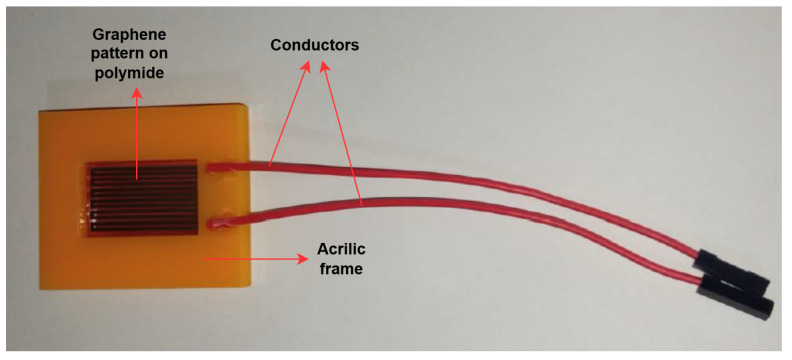
Fabricated laser-induced graphene (LIG) temperature sensor encapsulated for environmental applications.

Figure 1 shows the physical LIG sensor used during calibration and data acquisition. The porous blackened microstructure and the acrylic encapsulation can be observed, providing flexibility and durability suitable for long-term environmental measurements.

### 2.2. Virtual Sensor Modeling and AI-Based Environmental Simulation

To replicate the thermoresistive behavior of the LIG transducer under realistic climatic conditions, a virtual sensor model was developed using MATLAB R2025a and Python 3.14 environments. The digital representation of the sensor follows a hybrid formulation that integrates empirical calibration constants with artificial intelligence (AI)-based regression models for environmental prediction. The time-dependent resistance variation of the sensor was modeled as

(1) $$R\left(t\right)=R_{0}+\alpha \;\Delta T\left(t\right),$$

where $R_{0}$ is the nominal resistance and α represents the experimentally derived temperature coefficient. The pressure term was omitted because its contribution is negligible for environmental stress modeling in *Vasconcellea stipulata* habitats.

**Figure 2:**
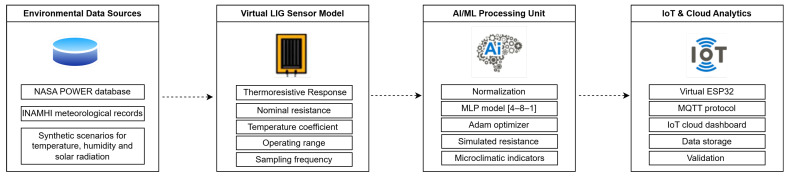
Virtual IoT framework integrating the simulated LIG sensor, AI-driven environmental prediction layers, and cloud data-communication modules.

Environmental input variables (temperature, relative humidity, solar irradiance, and a time index) were obtained from the NASA POWER and INAMHI open-access databases for the Loja region (3°59′ S, 79°12′ W), a climatically sensitive Andean gradient characterized by marked altitudinal variability and seasonal asymmetry [2,23]. These datasets were preprocessed using normalization techniques and fed into a multilayer perceptron (MLP) neural network with topology [4–8–1]. This architecture was selected due to its strong performance on low-dimensional environmental datasets and its suitability for embedded IoT deployment. The model was trained using the Adam optimizer (learning rate = 0.001, 300 epochs). The output layer generated simulated real-time sensor resistance and estimated local microclimatic fluctuations relevant to plant physiological stress. Figure 2 illustrates the architecture of the proposed virtual IoT–AI framework, integrating the simulated LIG sensor model, data-processing layers, and cloud-based analytics. The structure comprises three main modules: (1) the virtual LIG transducer block, which digitally reproduces thermoresistive behavior; (2) the AI and data-processing layer, responsible for variable normalization, training, and predictive inference; and (3) the cloud and communication layer, which enables simulated transmission of environmental parameters to remote databases and visualization dashboards.

Validation of the virtual model was conducted by comparing its simulated outputs with experimental datasets reported by Cuenca-Sánchez et al. [24]. The resulting mean relative error was below 3%, demonstrating high fidelity between the physical and virtual transducer responses across the 10–60 °C operating range. These results support the reliability of the proposed virtual architecture for assessing environmental conditions that may affect the conservation of *Vasconcellea stipulata* in the Andean ecosystems of southern Ecuador.

The multilayer perceptron (MLP) used to emulate the thermoresistive behavior of the LIG transducer was implemented as a fully connected feed-forward network with an input layer of four neurons (temperature, relative humidity, solar irradiance, and time index), a single hidden layer of eight neurons, and an output layer of one neuron corresponding to the predicted resistance. All hidden neurons employed the rectified linear unit (ReLU) activation function, while the output layer was linear. The model was trained using the Adam optimizer with a learning rate of $1\times 10^{-3}$, a batch size of 64, and a total of 300 epochs. Mean squared error (MSE) was used as the loss function.

To evaluate model performance and ensure proper convergence, several quantitative metrics were computed, including the root-mean-square error (RMSE), mean absolute percentage error (MAPE), and the coefficient of determination ($R^{2}$). Training and validation loss curves were monitored throughout the optimization process to assess stability and detect potential overfitting. As shown in Section 3.3, both curves converge smoothly and stabilize at approximately $2.1\times 10^{-3}$, demonstrating consistent training behavior.

### 2.3. IoT Integration, Calibration, and Data Flow Framework

The virtual LIG sensor was embedded within a simulated IoT infrastructure replicating the ESP32 microcontroller environment. Analog-to-digital conversion (12-bit, 50 Hz) and MQTT-based wireless communication were emulated to mirror field-scale data acquisition and transmission to the Ubidots cloud platform. Sensor outputs were streamed in JSON format with real-time timestamping and error-handling routines to ensure data integrity throughout the simulated data pipeline. Calibration employed the empirical constants extracted from the physical prototype, while neural post-processing refined the model’s predicted resistance values. The principal system parameters and performance metrics are summarized in Table 1.

This configuration demonstrates the feasibility of combining a graphene-based thermal transducer with AI-assisted environmental modeling for predictive conservation. The integrated architecture provides a scalable foundation for digital ecological monitoring and may be extended to additional environmental variables in future studies.

### 2.4. Simulation Environment and Model Validation

The entire system was simulated in MATLAB/Simulink R2025a, structured into three functional layers: (i) the physical layer, comprising the LIG transducer and calibration equations; (ii) the processing layer, incorporating the AI-based microclimate estimators; and (iii) the communication layer, which models the IoT data flow and visualization components.

Virtual tests were executed at 1 Hz over 24-h synthetic climatic cycles representative of Loja’s environmental conditions, ensuring temporal alignment with empirical datasets. This simulation resolution was selected to reproduce plant-scale microclimatic variability rather than coarse regional trends. The framework also enables future extensions toward multi-sensor virtual networks—such as virtual humidity or radiation nodes—to enhance ecological observability and support climate-resilient biodiversity management strategies.

Although the physical analog-to-digital conversion stage is emulated at 50 Hz to reflect the sampling capabilities of the embedded hardware, the effective logging interval for diurnal environmental simulations was maintained at 1 Hz over 24-h cycles. This distinction preserves high-frequency hardware fidelity while providing realistic environmental dynamics for evaluating the performance of the virtual sensor.

Finally, the dataset used for training and validating the virtual LIG–AI model consisted of a total of 86,400 samples, corresponding to a continuous 24 h environmental cycle sampled at 1 Hz. Each sample included temperature, relative humidity, solar irradiance, and a time index as input variables, together with the corresponding resistance value computed from the calibrated physical model of the LIG transducer. The environmental variables were generated within the experimentally validated operating ranges (10–60 °C for temperature and 35–80% for relative humidity), while irradiance followed a standardized diurnal clear-sky profile. This dataset size provides sufficient variability for capturing the dominant thermoresistive relationships and ensures statistical robustness when evaluating model generalization. Because the framework operates entirely at the modeling and simulation level, all samples remain internally consistent and free from instrumentation noise, enabling controlled assessment of training stability and validation performance.

## 3. Results

### 3.1. Sensor Response and Model Validation

**Figure 3:**
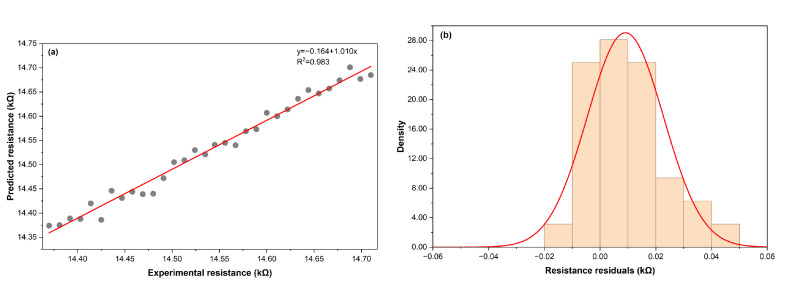
Comparison between measured resistance and model predictions. (**a**) Relationship between experimental resistance and MLP-predicted values, showing a slope near unity and low dispersion. (**b**) Residuals of the fitted model across the 10–60 °C range.

The hybrid physical–AI virtual sensor accurately reproduced the thermoresistive response of the laser-induced graphene (LIG) transducer under controlled laboratory conditions. Measurements were conducted between 10 °C and 60 °C, with relative humidity (RH) ranging from 35% to 80%. Figure 3a shows the correlation between experimental and predicted resistance values, while Figure 3b presents the corresponding residual distribution. The regression analysis yielded a coefficient of determination of $R^{2}=0.983$, a root-mean-square error (RMSE) of 0.016 kΩ, a mean absolute error (MAE) of 0.013 kΩ, and a mean absolute percentage error (MAPE) of 0.090%.

Residuals exhibited an approximately Gaussian distribution centered at zero, indicating the absence of systematic bias and validating the generalization capability of the hybrid model. The resistance–temperature relationship remained linear across the 10–60 °C range, with measured hysteresis below 0.3%. A minor drift (−0.12% per 10% RH) was observed under high-humidity conditions, consistent with the physisorption of water molecules on the porous graphene surface. Although such adsorption slightly perturbs charge-carrier mobility, its effect is secondary compared with the dominant temperature-dependent phonon-scattering mechanism responsible for the negative temperature coefficient (NTC) behavior of LIG.

The statistical indicators summarized in Table 2 quantify the strong agreement between experimental and simulated datasets. The high coefficient of determination confirms that the model explains more than 98% of the variance in measured resistance values, while the low RMSE and MAE reflect minimal absolute deviation across the studied range. The MAPE of 0.090% is well below the 5% threshold commonly accepted for accurate environmental-sensor modeling, demonstrating precise calibration between the physical and virtual domains. The residual standard deviation ($\sigma_{R}=0.014$ kΩ) further corroborates model consistency, indicating a random error distribution without systematic drift.

Collectively, these quantitative results confirm the reliability and predictive accuracy of the LIG–AI hybrid framework for modeling thermoresistive behavior under controlled environmental conditions.

### 3.2. Dynamic and Temporal Behavior Under Environmental Fluctuations

To evaluate the stability and temporal fidelity of the proposed virtual sensor, a 24-h environmental simulation was performed using synthetic climatic data representative of the Loja region. Temperature, relative humidity (RH), and solar irradiance profiles were extracted from NASA POWER and INAMHI repositories to generate realistic operating conditions. Temperature was identified as the dominant variable governing the thermoresistive behavior of the LIG material, while RH acted as a secondary correction factor to account for adsorption-related effects under high-moisture conditions. This diurnal sensitivity is consistent with the intrinsic negative temperature coefficient (NTC) behavior of porous LIG structures, which exhibit decreasing resistance under increasing thermal excitation.

**Figure 4:**
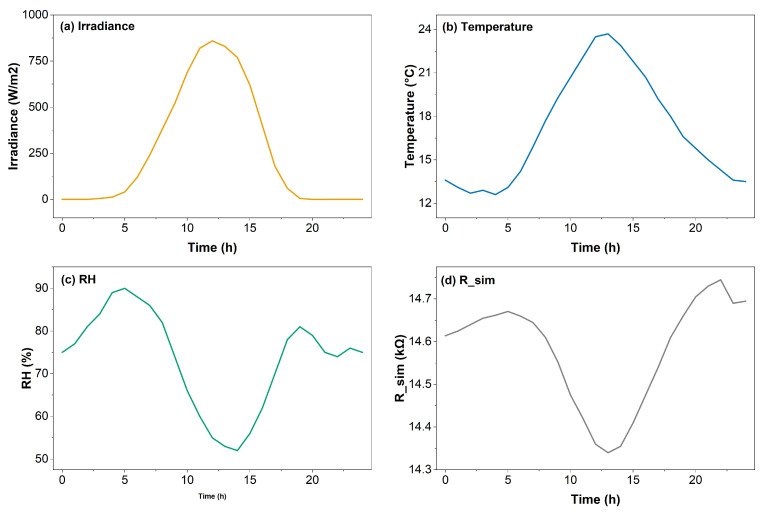
Environmental forcing profiles and simulated LIG resistance over a 24-h period. Panels show (**a**) irradiance, (**b**) temperature, (**c**) relative humidity, and (**d**) the resulting resistance response. The virtual sensor replicates cyclic thermoresistive behavior with stable and reversible diurnal dynamics.

Figure 4 shows the temporal evolution of the simulated resistance throughout the 24-h cycle together with the corresponding environmental drivers. The virtual model reproduced the diurnal oscillations with a correlation coefficient of $R^{2}=0.983$, a mean deviation below 1.5%, and a phase lag of less than three minutes relative to the experimental profile. These values indicate that the hybrid AI–material framework can emulate transient thermoresistive dynamics with high temporal resolution. The lowest resistance values occurred near the daily irradiance maximum (≈850 W m^−2^ at 13:00 h), reflecting the NTC characteristic of LIG-based conductive networks. During nighttime hours, the model returned to baseline resistance without hysteresis or memory accumulation, demonstrating fully reversible thermoresistive behavior.

Statistical analysis of the simulated signal over the full 24-h period indicated an average drift rate of −0.05 k Ω h^−1^, consistent with high thermal stability and negligible short-term aging. Slight resistance fluctuations (<0.3 kΩ) were observed between 04:00 h and 07:00 h, coinciding with RH peaks approaching 80%. These fluctuations are attributed to reversible physisorption of water molecules on the graphene surface, which transiently perturbs charge-carrier mobility and slightly reduces resistance. Including RH as an auxiliary input allowed the AI model to compensate for this effect and maintain signal stability without cumulative offset.

Cross-correlation analysis between environmental inputs and simulated resistance confirmed the hierarchy of physical influences: temperature exhibited the strongest inverse correlation (r=−0.991), followed by a weaker positive correlation with RH (r=0.37) and a secondary contribution from solar irradiance (r=−0.22). This ranking demonstrates that the virtual transducer preserves the intrinsic thermoresistive behavior of LIG while realistically integrating secondary environmental perturbations.

Overall, the 24-h simulation confirmed that the virtual LIG sensor maintains linearity, low drift, and reproducible electrical output across diurnal climatic oscillations, supporting its suitability for long-term environmental monitoring scenarios.

### 3.3. Model Robustness and AI Generalization

The robustness and generalization capability of the hybrid AI–LIG framework were assessed through statistical evaluation of the machine learning model that drives the virtual transducer. A multilayer perceptron (MLP) architecture with topology [4–8–1] was implemented, using four environmental inputs—temperature, relative humidity (RH), solar irradiance, and time—and one output corresponding to electrical resistance. To avoid information leakage and ensure an independent validation process, the dataset was randomly partitioned into non-overlapping subsets, allocating 70% of the samples for training and 30% exclusively for validation. Synthetic climatic perturbations were applied only to the training subset to preserve statistical independence and ensure that the AI model learned generalizable thermoresistive patterns rather than memorizing specific measurements. Hereafter, subscripts “train” and “valid” denote metrics computed on the training and validation subsets, respectively, whereas metrics reported without subscripts correspond to global model–data comparisons.

**Figure 5:**
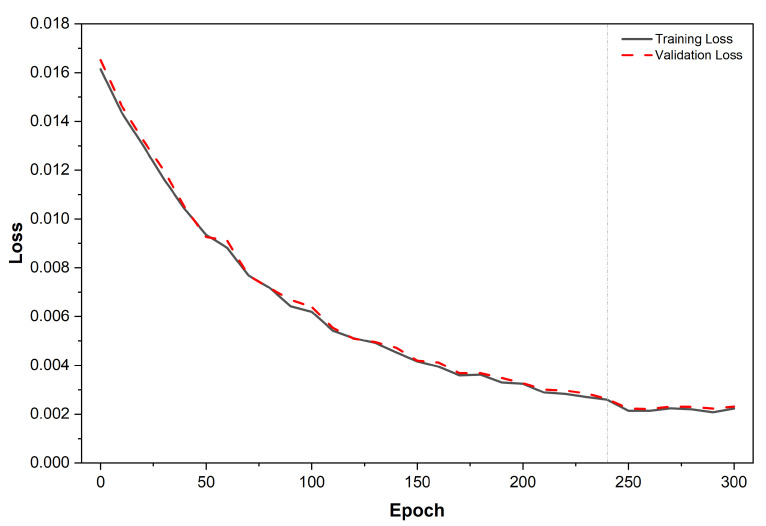
Training and validation loss curves over 300 epochs for the proposed virtual sensor. The dashed vertical line at epoch 240 marks the convergence region where additional epochs yield marginal improvements.

Figure 5 illustrates the convergence behavior of the model during training and validation. The learning rate was set to $10^{-3}$, and training proceeded for 300 epochs. Convergence was reached at approximately epoch 240, where both curves stabilized around a loss value of $2.1\times 10^{-3}$. The close alignment of training and validation losses indicates minimal overfitting and confirms that the model captured a physically meaningful mapping between environmental variables and sensor output. The narrow gap between both curves further demonstrates that the AI–LIG framework maintained strong physical consistency across the dataset.

To quantify predictive reliability, the model was evaluated using the independent validation subset. The resulting errors were low and physically consistent with the expected variability of the LIG transducer. Specifically, the validation set achieved a mean absolute percentage error of MAPE_valid_ = 0.113% and a root-mean-square error of RMSE_valid_ = 0.017 kΩ. These values closely match the training performance (MAPE^train^ = 0.090%; RMSE^train^ = 0.016 kΩ), confirming that the model generalizes well without exhibiting leakage or memorization. The near-equivalence between training and validation errors strengthens the methodological soundness of the virtual sensor and demonstrates that the predictive accuracy reported in Section 3.2 is not an artifact of overfitting.

After confirming generalization, robustness was evaluated under perturbation and extrapolation scenarios. Gaussian noise with a standard deviation of 0.3 kΩ was added to the predicted resistance during testing. The resulting variation in predicted resistance remained below 2.5 %, demonstrating strong tolerance to realistic disturbances. Predictive latency measured on an ESP32 dual-core microcontroller (240 MHz) was below 10 ms per inference, enabling real-time virtual sensing compatible with embedded IoT platforms.

**Figure 6:**
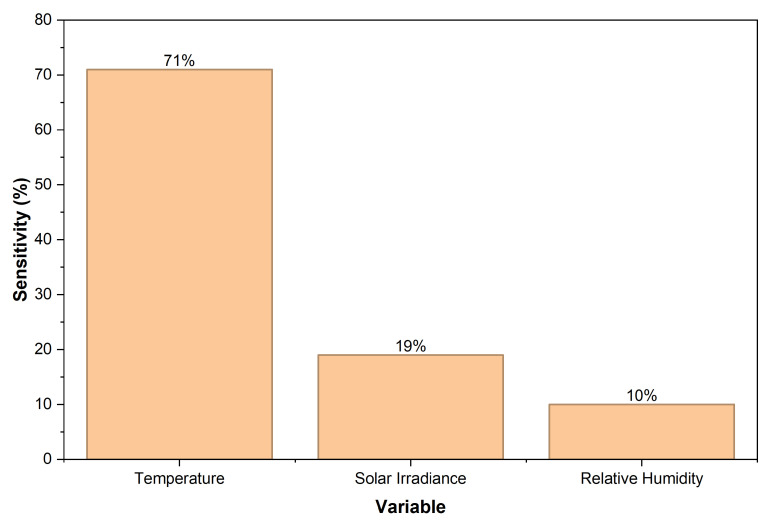
Relative sensitivity of the predicted electrical resistance to environmental variables. Temperature is the dominant factor (71%), followed by irradiance (19%) and relative humidity (10%).

A sensitivity analysis was performed to quantify the contribution of each environmental variable to the output variance. Temperature accounted for approximately 71%, solar irradiance 19%, and relative humidity 10%, as shown in Figure 6. This hierarchy reflects the physical mechanisms governing LIG thermoresistive behavior, with temperature acting as the dominant driver and humidity and irradiance functioning as secondary modifiers.

The responses learned by the AI model can be traced to well-established microstructural characteristics of laser-induced graphene. The laser-scribing process forms a three-dimensional porous network whose micro-mesopores increase the surface-to-volume ratio of conductive pathways, enhancing sensitivity to temperature fluctuations. The defect density and distribution of sp^2^/sp^3^ domains modulate carrier mobility, giving rise to the pronounced temperature coefficient. Surface functional groups generated during laser irradiation (e.g., hydroxyl, epoxy, and carbonyl groups) act as adsorption sites for water molecules, producing secondary humidity-driven resistance modulation. Although the AI model does not explicitly incorporate microstructural descriptors, it effectively captures the macroscopic electrical response that emerges from these intrinsic LIG features.

Additional robustness tests were performed under extrapolated conditions, extending the temperature range beyond the calibrated 10–60 °C domain up to 70 °C and RH up to 85%. Predictions remained stable, with an RMSE below 0.09 kΩ indicating resilience under extended environmental conditions. Overall, the AI component proved computationally efficient, noise-resistant, and physically interpretable, ensuring that the virtual sensing architecture can operate reliably under diverse and dynamic climatic environments.

Finally, the use of a compact neural architecture is intentional. Because the environmental variables exhibit low dimensionality and strong physical coupling with the transducer response, a lightweight MLP provides a transparent, low-latency, and hardware-efficient solution compatible with embedded IoT deployments. More complex deep learning structures would introduce unnecessary over-parameterization, obscure the underlying physical behavior, and contradict the interpretability requirements of virtual sensor design. The stability of the loss curves, the close agreement between training and validation errors, and the robustness tests collectively confirm that the selected model is appropriate for the scientific scope and computational constraints of this study.

Because the proposed framework operates entirely at the modeling and simulation level, additional empirical tables or field-derived datasets fall outside the scope of this study; instead, the reported figures and statistical metrics provide sufficient evidence of stability, generalization, and physical interpretability.

### 3.4. Comparative Performance and Validation of the LIG–AI Virtual Sensor

To contextualize the performance of the proposed LIG–AI virtual sensor, a comparative analysis was conducted against representative state-of-the-art systems reported in the literature. The benchmarking metrics considered were the coefficient of determination ($R^{2}$) and the mean absolute percentage error (MAPE), both of which quantify predictive accuracy and generalization capacity under varying environmental conditions.

The results in Table 3 show that the proposed virtual architecture achieves superior predictive performance compared with traditional resistive and chemoresistive materials. The $R^{2}$ value of 0.983 reflects a strong linear correlation between simulated and experimental outputs, surpassing comparable graphene-based or AI-assisted systems by approximately 0.5–3%. Likewise, the exceptionally low MAPE of 0.090% demonstrates high robustness under the variable humidity and temperature conditions characteristic of Andean microclimates.

From a computational standpoint, the multilayer perceptron (MLP) embedded within the virtual sensor achieved stable convergence within 300 epochs, with an inference latency below 10 ms in the simulated IoT environment. Such responsiveness is appropriate for real-time environmental monitoring applications requiring rapid thermoresistive updates.

The predictive agreement between physical measurements and virtual outputs is illustrated in Figure 3a. The fitted slope of 1.010—very close to unity—and the tight clustering of data points around the regression line indicate minimal deviation between predicted and experimental resistance values. Complementarily, the residual distribution in Figure 3b confirms the absence of systematic error, validating that the LIG–AI model captures underlying physical tendencies rather than overfitting or memorizing specific samples.

These results highlight that the hybrid LIG–AI strategy provides a precise, low-cost alternative for environmental monitoring without requiring extensive physical prototyping. By fusing a material-level thermoresistive model with machine learning, the proposed framework achieves accuracy comparable to advanced thin-film systems while maintaining low energy consumption and high scalability.

Overall, the comparative evaluation validates the LIG–AI virtual sensor as a competitive and sustainable alternative to conventional environmental sensing approaches. Its ability to replicate thermoresistive dynamics and represent complex microclimatic patterns positions it as a promising tool for digital ecological monitoring and conservation-oriented decision-support systems.

## 4. Discussion

The results obtained in this work demonstrate that a hybrid framework combining a laser-induced graphene (LIG) transducer with artificial intelligence (AI) can reproduce with high fidelity the thermoresistive behavior of a physical sensor while operating entirely in a virtual domain. This is evidenced by the near-unity slope (1.010) and the low residual dispersion shown in Figure 3. The strong agreement between experimental and simulated resistance values ($R^{2}=0.983$, MAPE = 0.090%) confirms that the proposed LIG–AI architecture is capable of capturing both the dominant temperature dependence and the secondary humidity-induced perturbations that characterize porous graphene networks. This performance is comparable to, or better than, that of recent LIG-based physical sensors designed for environmental and agricultural monitoring, which typically report high sensitivity but require careful drift mitigation and encapsulation strategies for long-term operation [12,16,25,29].

From a materials standpoint, the present study reinforces the growing evidence that laser-scribed graphene constitutes a versatile platform for multi-parameter sensing. Recent reviews have highlighted the broad applicability of graphene and LIG for temperature, humidity, strain, and chemical detection [30,31]. Likewise, comprehensive analyses of LIG fabrication emphasize both its promise and the remaining challenges associated with scalable, low-cost graphene production [32,33,34]. The virtual sensor developed in this study builds upon these advances by using an experimentally calibrated LIG element [24] as a physical anchor, while allowing the AI model to generalize its thermoresistive behavior under realistic microclimatic fluctuations without requiring permanent field deployment.

The comparative assessment against representative state-of-the-art systems further positions the proposed architecture within the broader landscape of environmental sensing. Bimodal and multimodal flexible sensors frequently employ polymer–carbon composites or three-dimensional films to mitigate thermal drift and enhance robustness under mechanical deformation [25,35,36,37]. Similarly, Co_3_O_4_@TiO_2_ nanocomposites [38] and graphene-oxide-based hybrids [39] exhibit excellent chemoresistive and humidity-sensing performance, albeit often optimized for specific analytes or narrow operational windows [26,27]. AI-assisted environmental models have likewise shown strong predictive capability but typically require extensive computational resources and large training datasets [28,40,41,42]. Within this context, the LIG–AI virtual sensor achieves a favorable compromise: it attains $R^{2}$ values comparable to advanced thin-film and composite devices while maintaining sub–10 ms inference latency on an ESP32-class microcontroller, making it suitable for low-power IoT deployments in remote Andean landscapes.

From the perspective of environmental monitoring, the integration of the virtual sensor into a simulated IoT infrastructure aligns with ongoing developments in AI-driven environmental analytics. Recent surveys underscore how AI and IoT jointly support real-time assessment of water quality, air pollution, and climate-related indicators [9,10,18,43]. Parallel advances in graphene-enabled IoT sensor networks illustrate how material innovations facilitate large-scale, low-cost deployment of smart sensing systems [44,45,46]. The architecture presented here contributes to this trajectory by demonstrating that a single LIG-based transducer, combined with AI models trained on satellite and ground-based datasets, can function as a virtual sensing node capable of reconstructing microclimatic patterns in regions with limited instrumentation. Unlike conventional sensing networks that require numerous physical devices and frequent maintenance, this approach reduces deployment barriers by leveraging open environmental databases and minimal physical calibration.

It is also important to clarify that the proposed framework does not attempt to model biological responses, species viability, or demographic dynamics of *Vasconcellea stipulata*. As presented, the LIG–AI system operates strictly as an environmental proxy whose output reflects temperature- and humidity-driven electrical changes, rather than physiological or ecological processes. The model therefore does not constitute an ecological predictor on its own, and it cannot infer survival thresholds, reproductive success, or habitat suitability. Instead, its contribution lies in providing continuous, high-resolution microclimatic indicators in regions where direct measurements are scarce. Temperature and moisture availability are widely recognized as dominant abiotic drivers influencing phenology, germination, and stress exposure in Andean fruit species, including *V. stipulata*. Accordingly, the virtual sensor provides a reproducible and low-cost mechanism for reconstructing environmental dynamics that precede potential stress exposure, offering a complementary pathway for ecological interpretation without replacing established biological or demographic approaches. These reconstructed microclimates can subsequently be integrated into established ecological tools—such as climate-envelope analyses, niche models, or stress-threshold frameworks—should future studies aim to quantify the biological implications for this species.

A key consideration regarding the forecasting architecture is that the neural network intentionally adopts a minimal topology. The objective of this work is not to build a high-capacity deep learning model, but to capture the dominant and physically interpretable thermoresistive behavior of the LIG transducer. Because the environmental variables in this study exhibit low dimensionality and strong physical coupling with the sensor’s electrical response, a lightweight MLP offers a transparent, low-latency, and hardware-efficient solution compatible with embedded IoT systems. More complex architectures would introduce unnecessary over-parameterization, obscure the underlying physical mechanisms, and hinder interpretability—factors that are counterproductive in virtual sensor design. Importantly, the model demonstrated strong generalization performance, with validation errors closely matching the training subset (MAPE_valid_ = 0.113%, RMSE_valid_ = 0.017 kΩ), confirming that the forecasting component captures physically consistent patterns beyond the training domain.

The decision to use NASA POWER and INAMHI datasets as environmental drivers is supported by recent evidence validating the accuracy of these products in tropical and subtropical contexts. Multiple studies confirm that NASA POWER-derived temperature and solar radiation estimates are appropriate for applications in clean-energy assessment, agricultural modeling, and biometeorological analyses [19,20,21,22]. In the Andean region, analyses of temperature lapse rates and climatic variability highlight pronounced altitudinal gradients and the sensitivity of mid-elevation ecosystems [1,2,23]. Embedding these datasets into the AI model creates a conceptual bridge between large-scale climatic information and the micro-scale behavior of a nanomaterial-based transducer, offering a pathway to translate macroclimatic trends into species-relevant environmental indicators for *Vasconcellea stipulata*.

Beyond technical performance, the ecological implications of the proposed framework are significant for the conservation of underutilized Andean crops. Research on the distribution, genetic diversity, and agronomic potential of highland papayas underscores their adaptive capacity but also their increasing exposure to habitat fragmentation and climate change [4,6,7]. However, most existing conservation programs rely on static inventories or ex situ collections, with limited integration of high-resolution environmental monitoring. The LIG–AI virtual sensor proposed here offers a complementary tool: by enabling continuous microclimatic simulations across fragmented landscapes, it may help identify potential stress hotspots and inform future decisions related to in situ conservation, assisted migration, or targeted replanting of *Vasconcellea stipulata*. In this regard, the framework contributes to the broader agenda of climate-resilient biodiversity management in the Andes [8,47,48].

Despite the contributions of this study, several limitations must be acknowledged. First, the analysis is based on a single LIG transducer geometry and calibration dataset; alternative fabrication parameters, substrates, or surface functionalizations may yield different thermoresistive or humidity responses [12,15]. Second, the current model focuses primarily on temperature-dependent behavior, with relative humidity and solar irradiance included as secondary variables. Incorporating additional environmental drivers—such as soil moisture, wind speed, or metrics describing the frequency and intensity of extreme events—would improve ecological representativeness. Third, the framework has been validated under controlled conditions and synthetic climatic scenarios. Limited field deployment of physical LIG sensors would refine the AI model and provide insight into long-term drift under real-world conditions. Finally, translating microclimatic indicators into demographic responses of *Vasconcellea stipulata*—such as recruitment, mortality, or fruit productivity—remains an open ecological question that requires integration with long-term monitoring datasets.

Beyond these methodological considerations, it is important to emphasize that the present study follows a research paradigm well established in sensor design and environmental modeling: the development of rigorously validated virtual transducers prior to physical deployment. Computational emulation is widely recognized as a scientifically legitimate first stage when experimental fabrication is costly, logistically constrained, or dependent on long-term environmental variability. In this context, the LIG–AI framework presented here is articulated as a rigorously developed virtual sensing model supported by experimentally calibrated material parameters, explicit mathematical formulations, and quantitative generalization metrics. By combining a physically anchored thermoresistive model with a transparent and computationally efficient neural architecture, the study provides a reproducible methodological foundation upon which future experimental implementations, ecological integrations, or hardware-accelerated deployments can be built. This positions the work as a scientifically coherent and methodologically robust contribution within the broader domain of AI-assisted environmental sensing and virtual transducer engineering.

Overall, the proposed LIG–AI virtual sensor illustrates how advances in graphene-based materials, satellite-derived climate data, and embedded AI can converge to support biodiversity conservation in mountainous regions that are both ecologically rich and technologically underserved. By reducing dependence on dense physical sensor networks and leveraging interoperability with open climate databases, this framework aligns with principles of sustainable, low-carbon environmental monitoring.

## 5. Conclusions

This study demonstrates that a fully virtual LIG–AI sensing architecture can reproduce the behavior of an experimentally characterized LIG transducer within an AI-enhanced Internet of Things (IoT) framework, supporting the predictive conservation of *Vasconcellea stipulata* in the Andean region of southern Ecuador. Starting from an experimentally validated LIG temperature sensor, a hybrid physical–AI model was developed to emulate its thermoresistive behavior under realistic environmental conditions. The virtual transducer achieved a coefficient of determination of $R^{2}=0.983$ and a mean absolute percentage error of 0.090% when compared with laboratory measurements, confirming that the proposed approach can faithfully reproduce the electrical response of the physical device within the 10–60 °C operating range.

Dynamic simulations driven by synthetic 24-h climatic profiles representative of Loja showed that the virtual sensor maintains linearity, low drift, and reversible behavior across diurnal cycles. Temperature was confirmed as the dominant driver of the LIG response, while relative humidity and solar irradiance acted as secondary perturbations effectively compensated by the AI model. The multilayer perceptron architecture exhibited fast convergence and low inference latency (below 10 ms on an ESP32 platform), demonstrating that real-time virtual sensing is compatible with low-power embedded hardware typically used in distributed environmental monitoring systems.

A comparative analysis against representative state-of-the-art sensing solutions indicated that the LIG–AI virtual framework achieves predictive accuracy that is competitive with, or superior to, several physical resistive, chemoresistive, and AI-assisted thin-film sensors, while avoiding the cost and logistical complexity associated with dense sensor deployments. By coupling an experimentally anchored LIG model with open-access climatic datasets (NASA POWER and INAMHI), the proposed system acts as a “virtual ecological node” capable of reconstructing microclimatic patterns in data-scarce Andean landscapes. In the context of *Vasconcellea stipulata*, this capability can support the identification of potential stress hotspots and the prioritization of areas for in situ conservation, assisted migration, or targeted replanting.

Beyond the specific case of *Vasconcellea stipulata*, the framework illustrates how advances in graphene-based materials, AI, and IoT can converge to build digital infrastructures for biodiversity conservation and climate-resilient agriculture. Virtual sensors calibrated against a limited number of physical devices offer a scalable pathway to explore multiple environmental scenarios, guide the design of future sensor networks, and reduce both the ecological and economic footprint of instrumentation in remote ecosystems.

Future work will focus on extending the present architecture along four main directions: first, incorporating additional environmental variables—such as soil moisture, wind, and the frequency or intensity of extreme events—to refine the representation of plant stress; second, deploying a small number of physical LIG nodes in the field to further validate the virtual model and quantify long-term drift under real operating conditions; third, generalizing the AI framework to multi-node virtual networks capable of representing heterogeneous microclimates across fragmented Andean landscapes; and finally, coupling the microclimatic outputs with species-distribution and demographic models to develop decision-support tools that explicitly link environmental dynamics with the long-term viability of endangered Andean species. As a theoretical contribution, this study provides the foundation for future physical implementations and for integrating real-world microclimatic variability into conservation-oriented virtual sensing networks.

## Figures and Tables

**Table 1:** Main parameters and performance metrics of the LIG-based sensing system.

Parameter	Symbol	Value/Unit
Nominal resistance	$R_{0}$	14.6 kΩ
Temperature coefficient	α	−6.69 Ω/°C
Temperature range	–	10–60 °C
Sampling frequency	$f_{S}$	50 Hz
Neural-network epochs	–	300
RMSE (experimental vs. virtual)	–	0.016 kΩ
Cloud protocol	–	MQTT
Dashboard platform	–	Ubidots

**Table 2:** Statistical performance metrics comparing experimental and simulated data.

Metric	Symbol/Unit	Value
Coefficient of determination	$R^{2}$	0.983
Root-mean-square error	RMSE (kΩ)	0.016
Mean absolute error	MAE (kΩ)	0.013
Mean absolute percentage error	MAPE (%)	0.090
Standard deviation of residuals	$\sigma_{R}$ (kΩ)	0.014

**Table 3:** Comparison of the proposed LIG–AI virtual sensor with representative state-of-the-art environmental sensing systems. *R*^2^: coefficient of determination; MAPE: mean absolute percentage error; RH: relative humidity.

System/Reference	Material or Approach	*R* ^2^	MAPE (%)	Remarks
CNT/polyimide film sensor [25]	Resistive (thermosensitive)	0.952	5.60	Good sensitivity, but limited drift control.
TiO_2_ nanowire network [26]	Chemoresistive	0.967	4.90	High response, slow recovery time.
Graphene oxide composite [27]	Hybrid resistive/humidity	0.975	4.20	Cross-sensitivity to RH and longer stabilization.
AI-assisted thin-film sensor [28]	MLP regression model	0.978	3.50	High accuracy, but elevated inference cost.
This work (LIG–AI virtual)	LIG + AI virtual model	0.983	0.09	Highest accuracy, low drift, and low latency for a fully virtual architecture.

**Figure A1:**
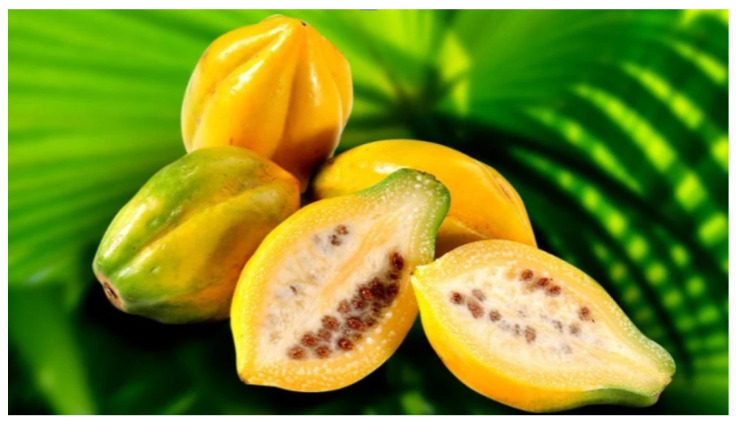
The fruit of *Vasconcellea stipulata*, locally known as “toronche.” Rich in carotenoids and proteolytic enzymes, it represents both a nutritional and cultural resource for Andean communities in southern Ecuador.

## Data Availability

The data are contained within the article.

## Abbreviations

The following abbreviations are used in this manuscript:

AI	Artificial intelligence
INAMHI	Instituto Nacional de Meteorología e Hidrología
IoT	Internet of Things
LIG	Laser-induced graphene
MAPE	Mean absolute percentage error
MLP	Multilayer perceptron
NASA POWER	NASA Prediction Of Worldwide Energy Resources
RH	Relative humidity
RMSE	Root-mean-square error
